# Idiopathic hyposmia as a marker of prodromal Parkinson’s disease — a cohort study

**DOI:** 10.1038/s41598-025-23293-4

**Published:** 2025-11-11

**Authors:** Zara Markovic-Obiago, Jonathan P. Bestwick, Harneek Chohan, Anette Schrag, Cristina Simonet, Alastair J. Noyce

**Affiliations:** 1 Centre for Preventive Neurology, Queen Mary University of London, London, UK; 2 Department of Clinical and Movement Neurosciences, University College London, London, UK

**Keywords:** Parkinson’s disease, Hyposmia, Prodrome, Olfaction, Neurology, Movement disorders, Parkinson's disease

## Abstract

Olfactory dysfunction is a well-established prodromal symptom of Parkinson’s disease (PD) and may occur years prior to PD diagnosis. This study assessed validity of a shortened 5-item smell test in a large cohort of PREDICT-PD participants and investigated its associations with previously described prodromal and demographic factors. Participants without PD aged over 60 years-old completed a 5-item smell test and online platform including validated questionnaires — such as the REM Sleep Behaviour Disorder Single-Question Screen (RBD1Q) and Unified Parkinson’s Disease Rating Scale (UPDRS) — as well as subjective olfaction assessment, and motor function assessment via the BRadykinesia Akinesia INcoordination (BRAIN) tap test. Log odds for PD were derived from smell test performance. Data was analysed by non-parametric tests in R-studio. A total of 1472 participants were included. Smell test performance declined with age and was poorer in males. Log odds for PD derived from the smell test correlated with RBD screening and bradykinesia as measured by BRAIN tap test but not motor impairment subjectively assessed by UPDRS part 2. Subjective olfactory assessments correlated weakly with objective smell test performance, with only fair agreement. Notably, subjective smell ratings did not correlate with motor function, whereas objective smell test results did. This study supports the feasibility of a shortened olfactory test for PD risk stratification in prodromal populations. Some previously described associations with prodromal and demographic factors were reaffirmed. Findings highlight the association between early motor dysfunction and olfactory impairment, emphasising the need for objective olfactory testing in research and clinical practice.

**Supplementary Information:**

The online version contains supplementary material available at 10.1038/s41598-025-23293-4.

## Introduction

Olfactory loss is a well-described symptom of prodromal and established Parkinson’s disease (PD), often predating clinical presentation by several years and present in up to 90% of established cases^1,2^. In apparently healthy older individuals, olfactory performance declines with advanced age and is poorer in males than females^3,4^. Some studies suggest that hyposmia may correlate with motor and non-motor symptoms in PD^5^.

In clinical contexts, patients are often asked to self-assess olfaction rather than using objective tools. The agreement between subjective and objective measures of olfaction is not robust^6^. In research settings, the most commonly used tools for testing smell are the University of Pennsylvania Smell Identification Test (UPSIT) and the Sniffin’ sticks. These tests are well validated but test length (40 and 16 scent items respectively), cost, and — in the case of Sniffin’ Sticks — the need for in-person supervised testing can limit their use in large-scale research or routine clinical objective olfactory assessment.

A shortened 6-item at-home smell test based on the items of the UPSIT common to both US and UK UPSIT versions most predictive of PD was previously developed by the PREDICT-PD team^7^. Answers to each scent are then used to generate information on a continuous scale, providing an extended range of likelihood ratios compared to dichotomous classifications of hyposmia/normosmia or total score of correctly identified scents alone. Shorter tests have the potential to offer faster and substantially more affordable olfactory testing^8^. Though abbreviated testing sacrifices comprehensive testing in favour of convenience and low costs, previous studies have suggested that short smell tests retain high sensitivity for hyposmia and PD detection^8,9^.

In this study, we assessed a cohort of PREDICT-PD participants’ performance using an abbreviated smell test and the correlation of shortened smell test performance with demographic and other prodromal factors. We also examined how subjective assessment of olfaction compared with performance on the shortened smell test.

## Methods

Participants without PD aged 60 to 80 years old living in the United Kingdom self-enrolled in the PREDICT-PD study (www.predictpd.com*)* and completed an online survey and a short microencapsulated odorant scratch-and-sniff smell test via the post. Four choices were available for each odour. Exclusion criteria were individuals below 60 years of age at the time of the smell test, those who reported neurological conditions (including stroke, dementia, restless leg syndrome, tremor), and those who self-reported having PD. The online survey collected demographic data on age, sex, and ethnicity. Validated questionnaires were included in the survey, including the REM Sleep Behaviour Disorder Single-Question Screen (RBD1Q), parts 1a, 1b, and 2 of the Unified Parkinson’s Disease Rating Scale (UPDRS), and the Hospital Anxiety and Depression Scale (HADS). Participants were asked to report if they had noticed a recent reduction in their sense of smell (responses were yes or no) and to rate their sense of smell on a scale of 0 (no sense of smell) to 10 (perfect sense of smell). Information collected on medical history included smoking history, previous head injuries causing loss of consciousness, bowel movements per week, history of erectile dysfunction, and family history of PD. Participants also completed the BRadykinesia Akinesia INcoordination (BRAIN) tap test, an online keyboard tapping test to assess upper limb motor function^10^.

A 6-item scratch-and-sniff smell test was posted to participants and self-administered at home. This 6-item test consisted of 6 odours previously proposed to be strongly associated with PD if mis-identified^7^. Predictive log odds and odds ratios for PD based on the PREDICT-PD model of PD risk in a general population were then calculated from the short smell test responses^7^. The petrol item was excluded from analysis of the smell test due to a manufacturing defect leading to an observed sudden increase in rates of misidentification in 2021 (see supplementary Fig. 1). The remaining 5 items (soap, watermelon, lemon, cinnamon, natural gas) were included in the final analysis. New coefficients for calculating predictive log odds, odds ratios, and likelihood ratios for PD based on the 5-item smell test were generated using multivariate logistic regression with the original data from which the 6-item smell test was calculated^7^. Model-based predictive risk of PD generated from participant 5-item smell test performance is referred to as “smell-test dependent log odds/odds ratio for PD” throughout the rest of the manuscript and is the primary smell test performance metric, giving the advantage of continuous data as the output^7^. The full PREDICT-PD algorithm risk score was not calculated. As the data were not normally distributed, non-parametric tests were used for statistical analysis, including the Kruskal-Wallis test, Mann-Whitney U test, and Spearman rank correlation coefficient. To assess adjusted relationships and control for covariates, we also fit multiple linear regression models adjusted for age. All statistical analyses were conducted in R Studio.

The PREDICT-PD study was approved by the Central London Research Committee 3 (reference number 10/H0716/85 and 13/LO/1457). All methods were performed in accordance with the relevant guidelines and regulations. All methods were conducted in accordance with the Declaration of Helsinki and the UK Policy Framework for Health and Social Care Research. Informed consent was obtained from all participants via the PREDICT-PD website.

## Results

Abbreviated smell test data were collected from 2,014 PREDICT-PD participants between 2018 and 2021. A total of 542 participants were excluded due to age < 60 years, incomplete data on the smell test date, self-reported PD, or self-reported other neurological conditions. Data on 1472 participants were included in the final analysis. Among those participants, 932 (63%) were male and 540 (37%) were female, with a mean age of 69 years old (SD = 5.34). Of the 369 participants who provided their ethnic background, only 8 (2%) identified as being from an ethnic minority group, leaving the study underpowered to assess associations between ethnicity and 5-item smell test performance.

Coefficients for the 5 items were generated from the same data from which the original 6-item test was generated, as in Table 1. Across the cohort, the median smell-test dependent log odds for PD were - 1.04 and ranged from - 3.09 to 6.8, SD 2.16, SE 0.06.

**Table 1:** 5-item smell test coefficients.

Odour	Co-efficient	95% Confidence interval
Intercept	6.797765	6.161798–7.433731
Soap	-2.31	-2.65- -1.98
Watermelon	-2.18	-2.54 - -1.81
Lemon	-1.71	-2.01 - -1.40
Cinnamon	-1.64	-1.96 - -1.31
Natural gas	-2.05	-2.45 - -1.65

The 5 odours present in both US and UK UPSIT proposed to be most associated with PD and the coefficients resulting from multivariate logistic regression^7^. Each coefficient is for the log odds for PD if an odour is correctly identified. Each correctly identified odour reduces the odds ratio for PD calculated from the 5-item smell test.

Males had an odds ratio of 1.60 (95% CI: 1.27–2.01) for PD compared with females (*p* = 1.08 × 10⁻⁵) based on smell test results. Smell-test dependent PD risk rose as age increased, with each additional year of age associated with a 9% increase in smell-test dependent odds ratio for PD (OR = 1.09, 95% CI: 1.07–1.11).

Smell-test dependent log odds for PD were not associated with having a first degree relative with PD (*p* = 0.11). No association was found with smoking history (883 never smokers, 569 ex-smokers, 20 current smokers, *p* = 0.53) or history of head injury causing loss of consciousness (1117 reported no history, 355 reported a history, *p* = 0.41).

**Fig. 1:**
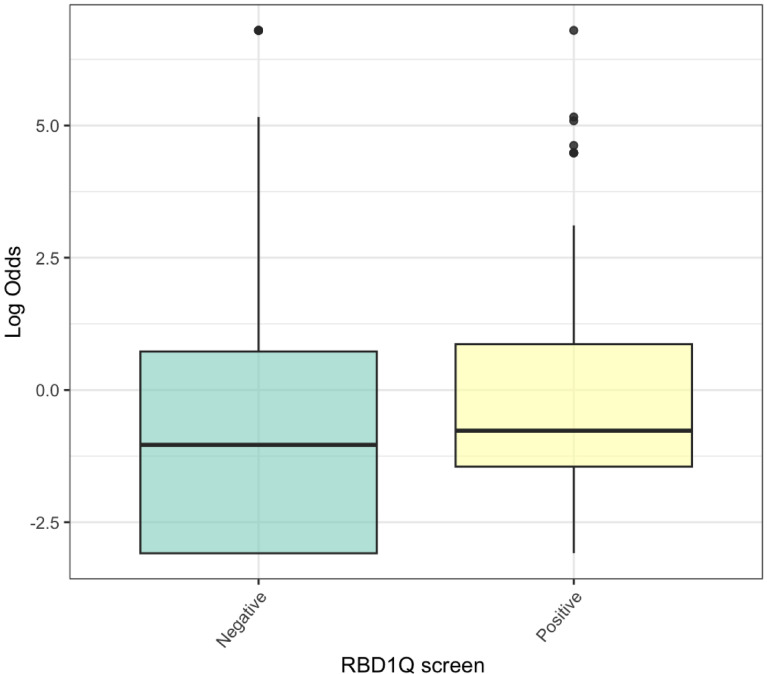
Relationship between RBD1Q screening for RBD and smell-test dependent log odds for PD based on the 5-item smell test. Participants were asked “Have you ever been told, or suspected yourself, that you seem to ‘act out your dreams’ while asleep?”. Those who answered “yes” had higher log odds, and therefore higher predicted probability of PD than those who answered “no”.

Fewer bowel motions per week was weakly associated with poorer smell-test dependent log odds for PD (median number of bowel motions per week = 4, *p* = 0.02). Each additional bowel motion was associated with an odds ratio of 1.09 (95% CI: 0.98–1.22), indicating a small and non-significant effect. There was no significant association with laxative use (*p* = 0.09). Those who screened positive for RBD on the RBD1Q had higher smell-test dependent log odds for PD (median log odds - 1.04 if answered “no”, median log odds - 0.77 if answered “yes”, *p* = 0.005) (Fig. 1). After adjusting for age, screening “yes” versus “no” was associated with an odds ratio of 1.74 (95% CI: 1.26–2.39). This indicates that participants who screened positive for RBD had significantly higher predicted PD risk, even after accounting for age. There was no relationship with Unified Parkinson’s Disease Rating Scale (UPDRS) 1a (*p* = 0.90), 1b (*p* = 0.36), or part 2 scores (*p* = 0.72). There was also no significant relationship with self-reported erectile dysfunction, use of medication for erectile dysfunction, or clinically significant HADS anxiety (*p* = 0.11) or depression (*p* = 0.11) scores.

**Fig. 2:**
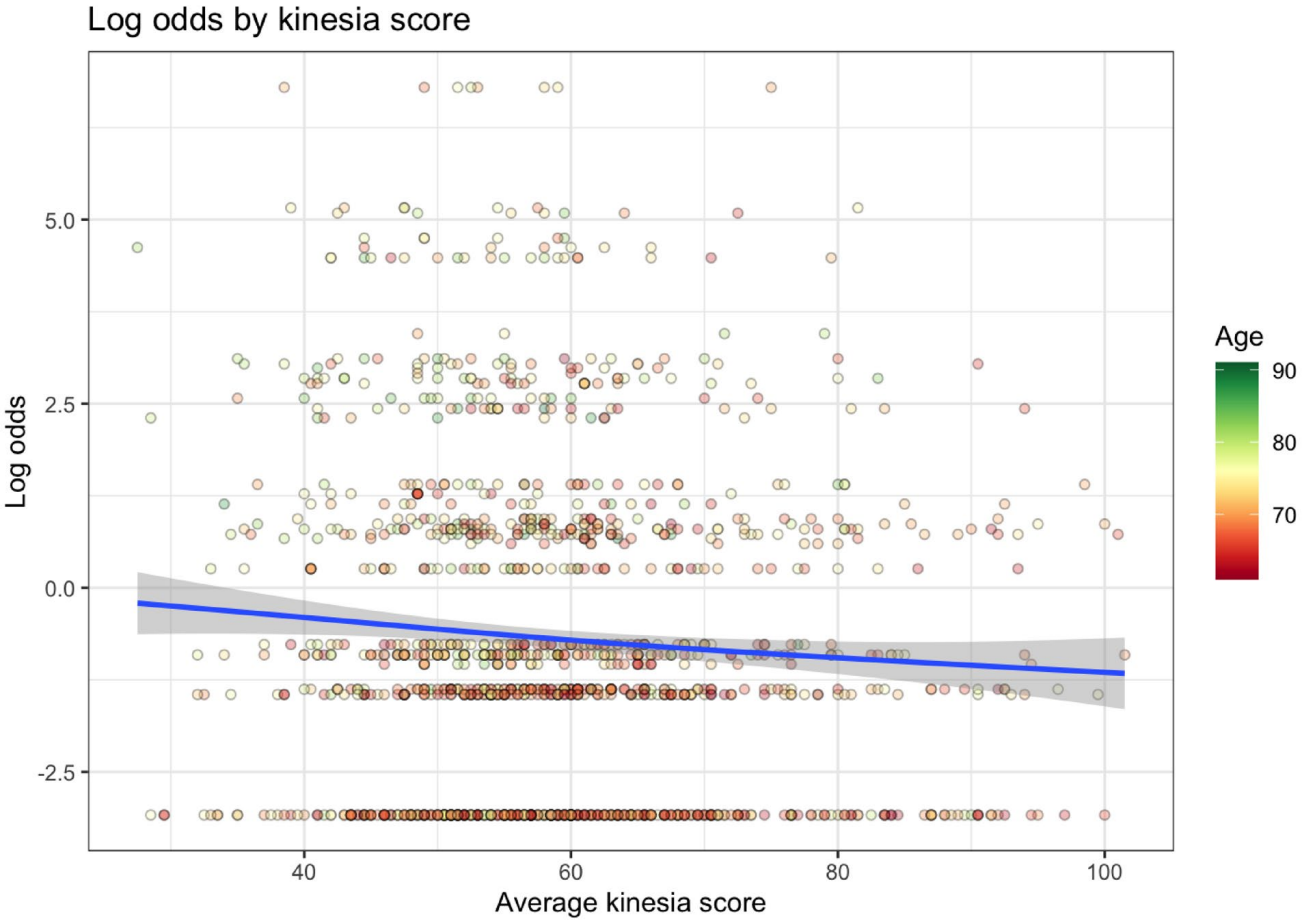
Relationship between smell-test dependent log odds for PD from 5-item smell test and average of left- and right-hand kinesia scores. Lower kinesia scores (indicating more bradykinetic tap tests) were associated with higher smell-test dependent log odds for PD from the 5-item smell test and higher smell test predictive probability for PD. Age is represented by the color of each data point (dot): red dots indicate younger participants, transitioning through yellow, to green, which indicates older participants.

Kinesia scores (KS) from the BRAIN tap test were calculated as the number of alternate taps in 30 seconds with the left and right hands. KS values below 15 or above 110 were excluded. KS were approximately normally distributed (mean 59.11, SD 12.40; median 57.50, IQR 51.0–65.50). In multiple linear regression adjusting for age, higher (better) KS were significantly associated with lower smell-test dependent log odds of PD (coefficient = -0.0097, *p* = 0.039, Table 2; Fig. 2). Expressed as odds ratios, each unit increase in KS was associated with an odds ratio of 0.990 (95% CI: 0.981–0.996). While the per-unit effect was modest, a 10-point increase corresponded to an OR of 0.91 (95% CI 0.83–0.995). Across the IQR (IQR 51.0–65.50), this corresponded to an OR of 0.87 (95% CI: 0.77–0.99), and across the 5th–95th percentile range (41.0–83.5), the OR was 0.66 (95% CI: 0.49–0.89). Average akinesia time (AT) was calculated as an average of cumulative time keys were depressed during the tap test with the right and left hand any time a key was depressed for longer than 17ms. Longer AT was associated with higher smell-test dependent log odds for PD from the 5-item smell test when assessed by Spearman’s rank correlation coefficient (*p* = 0.009), but not when the relationship was adjusted for age in multiple linear regression (Supplementary Table 1).

**Table 2:** Multiple linear regression model of smell-test dependent log odds for PD, kinesia scores, and age at smell test.

Average kinesia score over left and right hands, mean KS = 59		
Variable	Coefficient	*P*-value
Intercept	-5.28	< 0.001
Kinesia score	-0.0097	0.039
Age at smell test	0.075	< 0.001

Coefficients and p-values for multiple linear regression model evaluating the relationship between smell-test dependent log odds for PD from 5-item smell test, age, and left- and righthanded kinesia scores respectively. Poorer average kinesia score is associated with higher smell-test dependent log odds for PD from 5-item smell test and therefore higher predicted risk of PD.

### Association of smell test performance and subjective smell rating

**Fig. 3:**
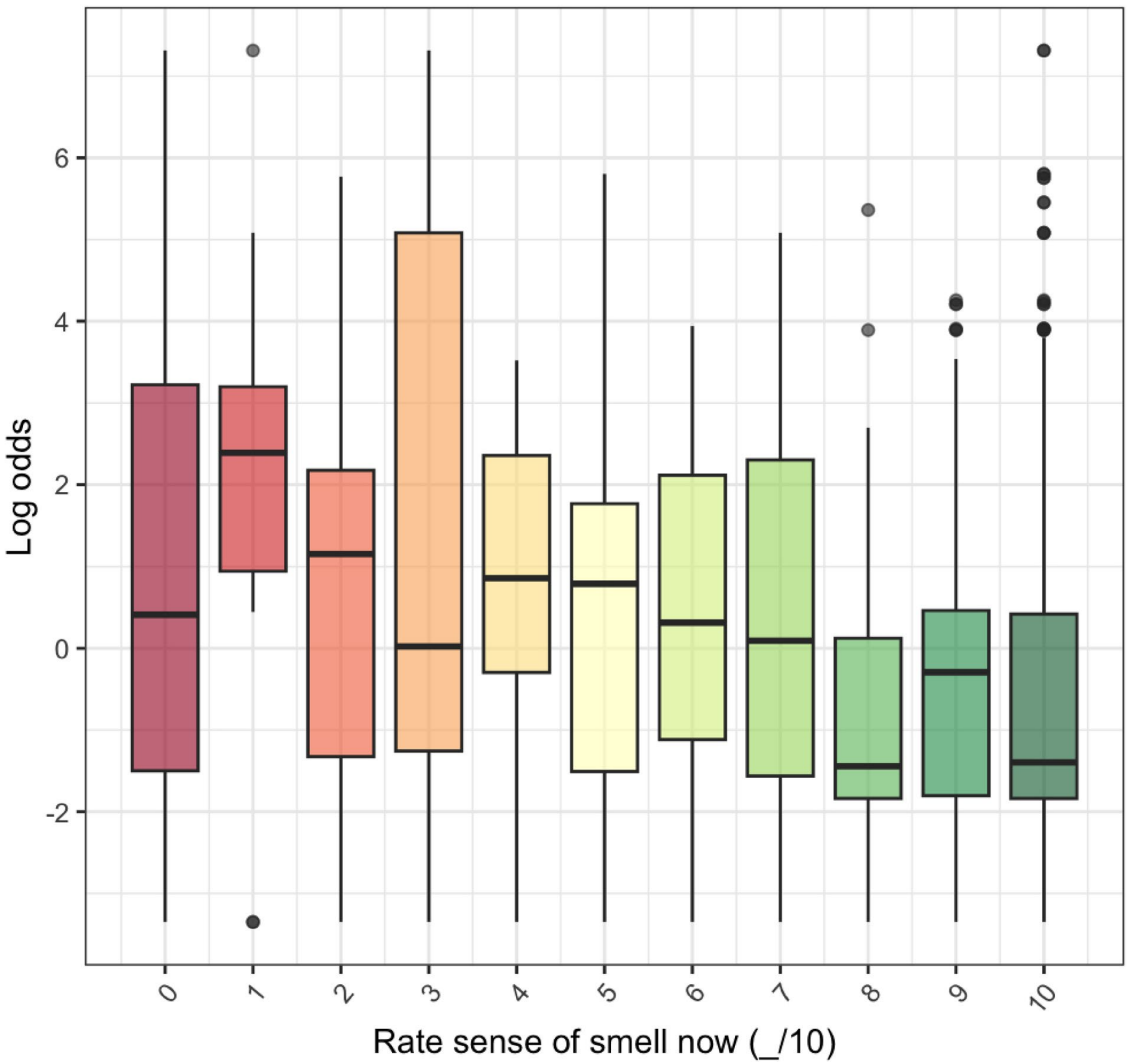
Relationship between subjective smell ratings (0–10) and smell-test dependent log odds from for PD the 5-item smell test. Higher smell-test dependent log odds represent a higher modelled probability of PD. Overall, those who rated their own sense of smell as poorer had a higher probability of PD from the 5-item smell test objective assessment.

**Fig. 4:**
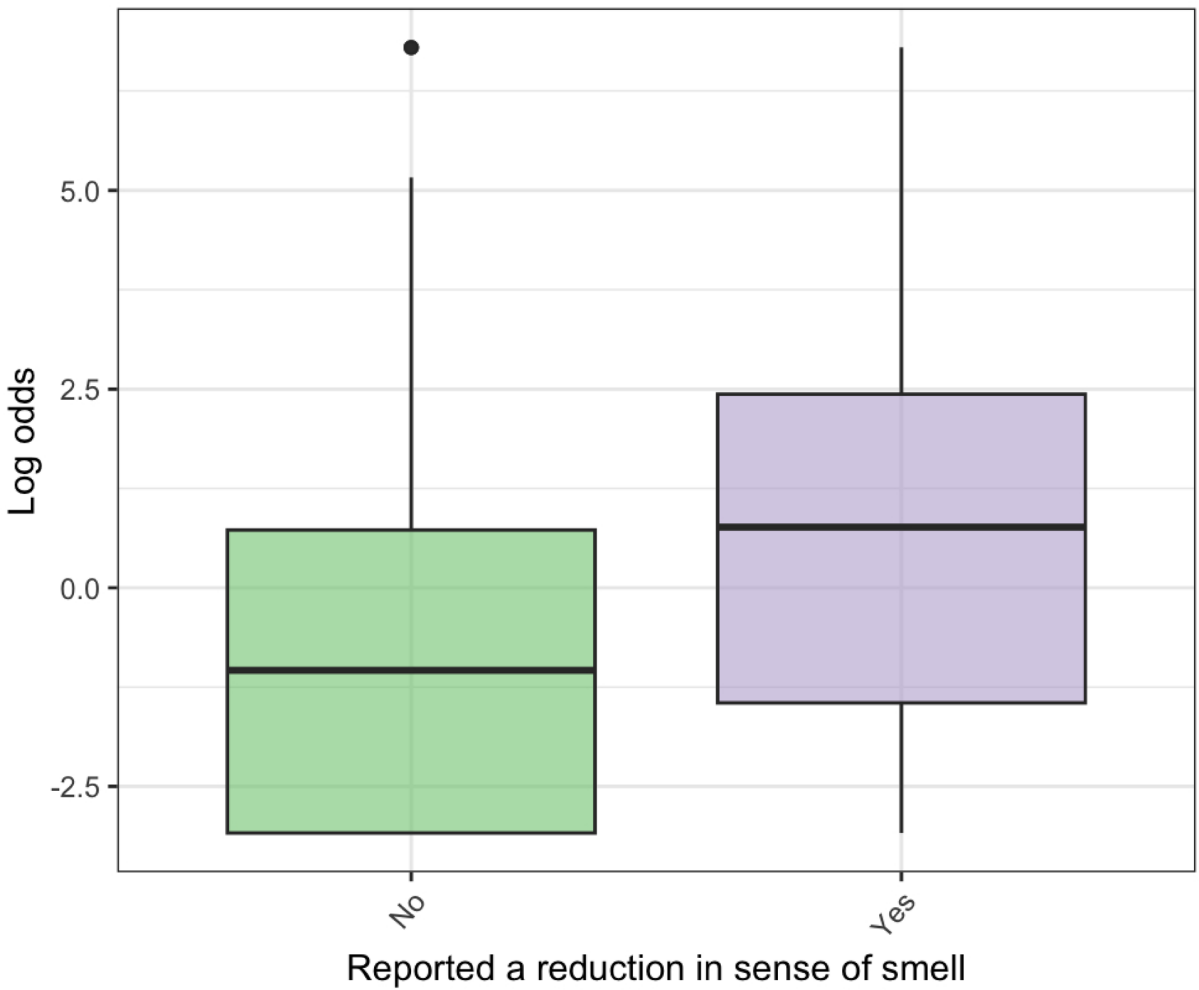
Relationship between reported reduction in sense of smell and smell-test dependent log odds for PD. Higher log odds for PD represent a higher probability of PD. Those who had noted a change in their own sense of smell performed worse on the 5-item smell test, reflected in higher smell-test dependent log odds for PD and a higher prediction of PD.

The majority of participants rated their smell as 10/10 (806 out of 1206, 67%) and there was significant evidence for an association assessed by Spearman’s rank correlation coefficient (rho = -0.15, *p* < 0.001) between subjective and objective assessment as measured by smell-test dependent log odds of PD (Fig. 3). Linear regression indicated that each 1-point increase in smell rating out of 10 was associated with a 19% fall in smell-test dependent odds of PD (OR = 0.81, 95% CI: 0.77–0.85). There was also a relationship between reported recent change in sense of smell and worse smell-test dependent log odds for PD from 5-item smell test performance (*p* < 0.01) (Fig. 4). However, agreement between the 15% of participants who rated their sense of smell the worst (subjective rating < = 7/10) and the 15% with the poorest 5-item smell test performance (smell-test dependent log odds for PD > = 1.40) was only fair (Cohen Kappa correlation coefficient of agreement = 0.24).

While there was a significant relationship between smell-test dependent log odds for PD from smell test performance and kinesia scores when adjusted for age, there was no significant relationship between subjective smell rating (on a scale of 0–10) and kinesia scores (Supplementary Table 2).

## Discussion

This study set out to validate the use of an abbreviated smell test in a large number of healthy controls and identify known and novel associations with more extensive testing. The study reiterates two of the most widely found associations with hyposmia: there was a significant association with sex, where males generated higher log odds for PD from the 5-item smell test than females, and with age, where olfactory performance measured by modelled log odds for PD became worse as age increased. However, there were notable absent associations with other factors. No significant association was found between smell-test dependent log odds for PD and smoking status, history of head injury causing loss of consciousness, or family history of PD. The absence of an association with smoking history may be due to low numbers of current smokers, particularly if many ex-smokers quit over 15 years prior to smell testing, allowing their sense of smell to normalise^11^.

Performance on the 5-item smell test was associated with RBD, a hallmark feature of prodromal PD, as measured by a positive screen for RBD via the RBD1Q. The relationship with constipation, another prodromal PD feature, was weak. Objective measurement of bradykinesia, as measured by KS on the BRAIN test, showed increased bradykinesia with worsened smell-test dependent log odds for PD. However, subjective assessment of bradykinesia via the UPDRS part 2 did not show any relationship, which may represent reduced sensitivity of this scale to assess for bradykinesia in a prodromal population, or a lack of awareness of motor signs. No significant association with non-motor symptoms of PD was found with UPDRS parts 1a or 1b. The lack of correlation may represent how complex olfactory dysfunction is in the context of PD risk in an unselected general population, particularly following the COVID-19 pandemic. The lack of associations may also stem from this 5-item smell test being tailored to scents predictive of PD risk rather than assessing general olfaction. This could alter the pattern of associations observed, though the existence of a PD-specific odour set is much debated^9,12^.

A significant relationship between motor dysfunction, measured by poor kinesia score on the BRAIN test, and poor shortened smell test performance, even when adjusted for age, emphasises the importance of olfactory testing. The large effect of 10-point change in KS demonstrates that realistic differences in KS are associated with appreciable differences in modelled PD risk. The presence of a relationship between shortened smell test performance and motor dysfunction, but not between smell test performance and most aspects of non-motor prodrome of PD, may also indicate that olfactory deficits occur in a different prodromal population who develop motor dysfunction versus a population with a heavier burden of non-motor prodromal features.

Most clinical settings in the UK do not perform objective testing for hyposmia. Our finding of only fair agreement between rating sense of smell (on a scale of 0–10) and 5-item smell test performance shows some alignment with subjective assessment of olfaction, but the imperfect agreement between subjective and objective measures of olfactory impairment argues strongly for routine use of objective testing to reduce the impact of smell agnosia in at-risk populations. The lack of significant association between olfaction assessment and motor dysfunction (via kinesia score) when olfaction is assessed subjectively, versus the presence of a significant association when olfaction is assessed objectively (via smell-test dependent log odds for PD), further demonstrates the crucial importance of objective hyposmia testing. Additionally, the presence of a relationship between objective bradykinesia measures (KS in BRAIN testing) and objective olfactory assessment (5-item smell-test dependent log odds for PD), but absence of a relationship with subjective olfactory assessment (rating sense of smell), emphasises a general need to test objectively for the other often subtle features of prodromal PD. A shorter smell test would allow collection of this diagnostically useful information while minimising time and financial constraints in a clinical setting.

Strengths of this study include the substantial sample size of those completing the online survey and shortened smell test. This study comprehensively explored widely used indicators of prodromal PD and used validated tools where possible, increasing the credibility of findings and the generalisability of the work to other studies. Assessing smell on a continuous scale allows for expanded ranges of likelihood ratios versus binary outcomes, allowing for more nuance in the relationship between olfaction and other factors to be explored. Compared to UPSIT or Sniffin’ sticks, this 5-item home test has the advantage of brevity, low cost, and remote administration without requiring trained supervision. While lower diagnostic utility with shorter smell tests has been proposed^9^, previous analysis showed the shortened smell test to have comparable performance to longer UPSIT testing^7^. Our findings support the utility of the short test as a practical, scalable tool for population risk assessment or for risk stratification in primary or secondary care where objective olfactory testing is not standard practice. This study extends the understanding of hyposmia by using a test angled towards modelled PD risk specifically, rather than olfactory impairment generally, which may give a disease-specific perspective on the relationship between hyposmia and demographic, past medical, and prodromal factors. Remote assessment of motor dysfunction via the BRAIN test allowed for differences in shortened smell test performance associations with motor and non-motor prodromal factors to be appreciated.

### Weaknesses

While data were collected on participant ethnicity, data were incomplete and this study was not sufficiently ethnically diverse to assess any association between performance on the 5-item smell test and ethnicity. Scent recognition and description have been proposed to vary across different ethnicities and cultures, and we should seek to assess performance of the test across different ethnic groups^13,14^, particularly given concerns that disease-specific shortened smell tests do not translate across different cultural cohorts^9^. This study focuses on the association with prodromal and demographic factors but lacks exploration of other lifestyle factors that could influence both PD risk and olfactory function, such as diet and environmental exposures. The voluntary self-enrolment of PREDICT-PD may also introduce some selection bias and impact the generalisability of the findings to the broader population. This study did not assess for cognitive impairment beyond question 1.1 of the UPDRS. While these model-derived risk scores provide valuable continuous estimates of PD risk, they do not confirm the presence of prodromal or clinical PD, and may allow for misclassification, underscoring the importance of further validation. The cross-sectional nature of the study limits the ability to draw conclusions on correlation or causation. A longitudinal study would also allow us to also capture how prodromal and demographic factors impact the rate of olfactory decline over time. The exclusion of the petrol item from the smell test unfortunately narrows the likelihood ratio range generated from the test. The manufacturer of the scents denied any change to the formulation that explained the sharp and sustained decrease in correct identification of petrol scent and increase in misidentification of the scent as peach over 2021 and 2022. Though it remains unclear why the identification of this scent became unreliable, its exclusion from the analysis of shortened smell test results was necessary for accuracy. Further investigation of quality control of olfactory tests over time is important.

### Future directions

Recognising these weaknesses is important for interpreting current results and guiding future olfaction studies. Some steps are already being taken to address these, such as current use of the shortened smell test in a highly ethnically diverse cohort of the East London Parkinson’s disease Project, a prospective study recruiting participants with and without PD to identify biomarkers, validate screening tools, and characterise PD within one of the most ethnically and socioeconomically diverse populations in the UK^15^.

## Supplementary Information

Below is the link to the electronic supplementary material.

Supplementary Material 1

## Data Availability

Data is not provided within the manuscript or supplementary information files due to restrictions on data sharing due to privacy concerns, ethical considerations, and participant confidentiality. Applications for PREDICT-PD data, which include intended scope of use, are reviewed by the PREDICT-PD steering committee (contact via Alastair J Noyce at a.noyce@qmul.ac.uk).
