# Supplementary material for: Idiopathic hyposmia as a marker of prodromal Parkinson’s disease — a cohort study
Source: Sci Rep. 2025 Nov 11;15:39501. doi: 10.1038/s41598-025-23293-4 (PMC12606346; doi:10.1038/s41598-025-23293-4)
Supplement: Supplementary file 1 — Supplementary Material 1 [file 41598_2025_23293_MOESM1_ESM.pdf]

Supplementary Material

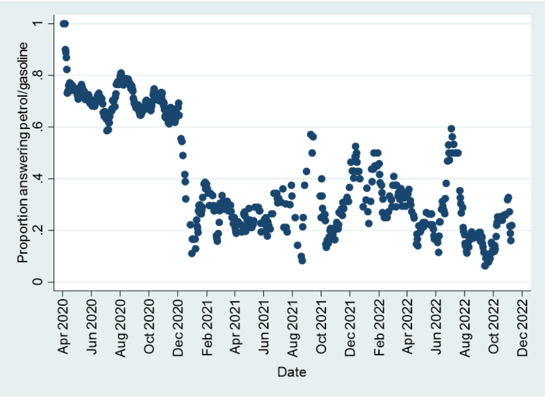

1a)

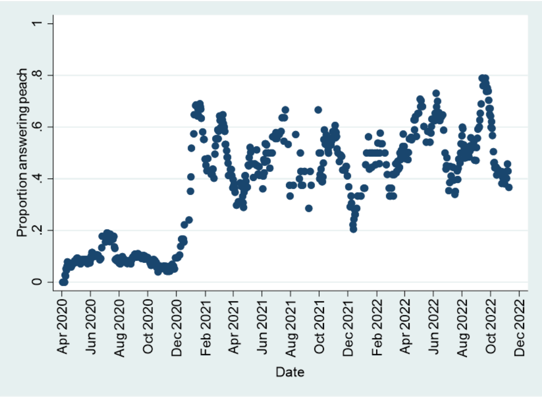

1b)

Supplementary Fig. 1: a) proportion of participants correctly answering petrol/gasoline and b) proportion of participants incorrectly answering peach

**Average akinesia time over left and right hands, mean = 97ms**		
**Variable**	**Coefficient**	**P-value**
Intercept	-6.57	<0.001
Akinesia time	0.003	0.11
Age at smell test	0.081	<0.001

Supplementary table 1 Coefficients and p-values for multiple linear regression model evaluating the relationship between log odds from 5-item smell test, age, and average akinesia time. Poorer average akinesia time does not have a significant relationship with log odds from 5-item smell test when adjusted for age

**Variable**	**Coefficient**	**P-value**
Intercept	8.40	<0.001
Average kinesia score	0.01	0.10
Age at smell test	-0.0032	0.822

Supplementary table 2 Coefficients and p-values for multiple linear regression models evaluating the relationship between subjective rating of sense of smell/10, age, and average kinesia scores. There is no significant relationship between subjective smell assessment and objective bradykinesia measures here
